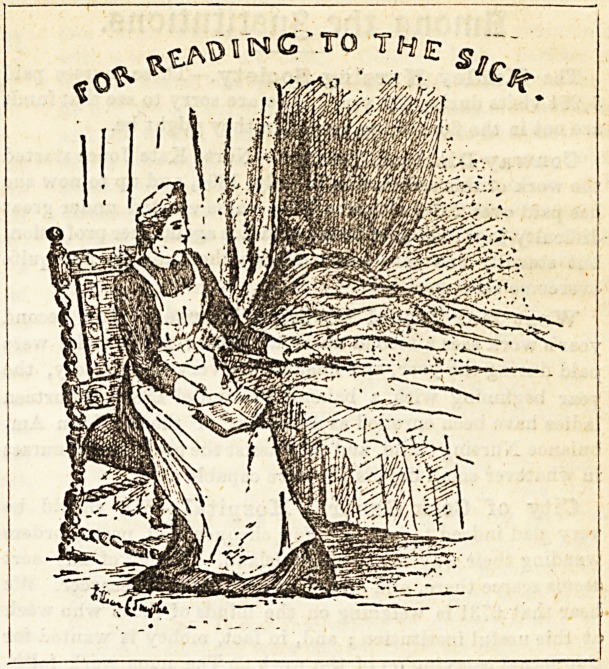# The Hospital Nursing Supplement

**Published:** 1892-05-21

**Authors:** 


"The Hospital" Nursing Mirror.

Being the Extra Nursing Supplement of "The Hospital" Newspaper.

Contributions for this Supplement should be addressed to the Editor, The Hospital, 140, Strand, London, W.C., and should have the word "Nursing" plainly written in left-hand top corner of the envelope.

### En Passant.

THE NURSES' CO-OPERATION.—The meeting of the Executive Committee of the Nurses' Co-operation took place on the 11th inst., and great regret was expressed at the resignation of the Hon. Treasurer, Dr. Goodhart, who has given so much practical proof of his interest in the undertaking since its commencement. The report of the financial position of the Co-operation was most satisfactory. General Ross was appointed to the office of Chairman, vacant through the retirement of Mr. Cheston.

THE LONDON TEMPERANCE HOSPITAL.—The public opening of the "Grosvenor" Children's Ward at the London Temperance Hospital, took place in the afternoon of May 11th. The ceremony was attended by a large number of visitors, who seemed greatly interested in the inmates of the pretty cots, 14 in number, and especially in two delicate babies lying in swinging cots in the centre of the tastefully decorated ward. The whole of the hospital was open for inspection, and the exquisite order of the nursing appliances, the brightness of the airy wards, the neatness of the convenient little kitchens were alike admirable. The nurses' uniforms are specially commendable, being in every respect suitable for work amongst the sick.

A DIET DISPENSARY.—So far we do not possess over here an establishment of this sort, and financially it sounds a trifle doubtful. Some ladies in St. Louis are, however, trying how they can make it succeed. New-laid eggs, jellies, beef tea, broth, and other invalids' dishes are to be kept always ready and they will be furnished to patients on their presenting a written prescription from a doctor as they would for medicine. Poor people cannot afford such things, could they even make them, and it is thought that rich people may be glad to avail themselves of the dispensary; and it is hoped that the payments of the rich will be sufficient to enable the poorer people to be supplied at the lowest cost.

DR. SUTTON AND HIS STUDENTS.—In one of his lectures to students, the late Dr. Sutton gave a certain piece of advice, which applies so exactly to the nurse of the present day, that if our readers will but change the words "Medical Man" into "Qualified Nurse," and the "he" into "she," the practical suitability of the lesson contained in the following passage is self-evident: "Don't underrate the influence of your own personality. Learn to give confidence to your patients. On his way to become a medical man the student has to pass through three stages: First he doesn't know; then, he thinks he knows; then he knows he doesn't know, but he stands on his feet like a man and gives confidence to his patients."

THE FADDISTS OF THE DAY.—Mr. Boyd Carpenter, in his speech at the meeting of the Metropolitan and National Nursing Association, made most appreciative mention of the change which takes place in a sick person's room when a trained nurse enters into possession, and earnestly advocated the need for an increased band of qualified workers, which ought not to be so difficult of attainment when the amount of energy wasted in London is considered. He spoke of the "mere faddist of the day," as contrasted with the earnest worker with very sound sense. We must, most of us, have in our minds instances of work commenced with enthusiasm and disastrously ended by those most incompetent of busybodies, the "dabblers in charity," and, worst type of all, the dabblers in sick nursing amongst the poor.

THE INSANE OF MONTENEGRO.—In spite of Prince Nikita's efforts at civilisation, Montenegro is as yet without one single public or private asylum. The insane roam at large or are kept with their families; if they become violent "bleeding" is resorted to; and when this fails they are shut up in a hut by themselves. The influential families are considered lucky when they can get permission to place their insane relative in a cell of the State Prison at Cettinje till death releases him from a terrible life. On this subject, which seems a nineteenth century impossibility, there is an article in *Good Words* for this month.

THE PROPOSED NURSING INSTITUTION AT BIRMINGHAM.—The working men of Birmingham and the indefatigable Secretary of the Hospital Saturday movement there have excelled themselves. A convalescent home is being provided with the balance over and above the amount which the Hospitals receive every year. Now the Committee is meditating a scheme for establishing a nursing institution from which nurses may be sent into the homes of the working classes to superintend, if nothing more, the nursing of the sick. There is nothing mean or small about the hopes of success among Birmingham folk; they hope to get £12,500 this year, and if this amount is raised £1,000 will go directly to the furtherance of the nursing scheme. It is a pity that enthusiasm as practical as this does not partake a little more of the nature of an epidemic.

NORTHERN WORKHOUSE NURSING ASSOCIATION.—The annual meeting of this society came at an opportune moment, for the subject of trained nurses for the sick poor at Leeds has just been under discussion by the guardians of that city, and we regret to hear they have appointed two new nurses who, though no doubt very estimable persons, are not trained nurses. Leeds seems to be a bit behind the times. One enlightened gentleman is said to consider trained nursing "pampering of the paupers." Poverty is a deadly sin in some eyes. We wonder if that gentleman has considered the fact that trained nursing shortens the stay of a patient in hospital; it is worth consideration from an economic point of view alone. The Association steadily gains ground and support, financial and otherwise; twenty nurses are in training at the various workhouses, and twenty-nine boards of guardians in the district subscribe to the funds. We hope the annual meeting, at which some admirable speeches were made, will be the beginning of a new education for Leeds guardians.

SHORT ITEMS.—The Duke and Duchess of Portland will open the bazaar on the 24th of this month at Swiss Cottage, in aid of the women's wards of the Great Northern Central Hospital.—Anything worse than the condition of Keighley Infirmary cannot be imagined, both as to accommodation and administration; there is plenty of reform work to be done in the north.—Rye is to have a district nursing association, modelled on Miss Broadwood's plan of having only partially trained nurses.—Dr. Janet Young Hunter, of the Free Church of Scotland Medical Mission, died last week in India; her career had promised to be most successful.—The Countess of Denbigh appeals for the Orphanage of the Sisters of Charity in Carlisle Place, Victoria Street; an outbreak of scarlet fever forbids their selling any needlework, which constitutes their principle means of livelihood.—A nurse writes to say what a pleasant holiday she has spent at the Kent House Holiday Home at Margate.

### Ventilation, Disinfection, and Diet.

By P. Caldwell Smith, M.D.

### VI.—DISINFECTION.

The Germ Theory—Germs the Cause of Putrefaction—Germs as the Causal Agents in Disease—Koch's Essentials—The Lower Fungi—Varieties of Fungi—True Fungi—Mycetozoa—Yeast Fungi—Fission Fungi or Bacteria—Micrococcus—Bacillus—Spirilla—Irregular Forms.

It is now a well-established fact that a large number of diseases, called zymotic or infectious diseases, are caused by micro-organisms or germs, and it is to destroy these that we require to use disinfectants. It is necessary to say a little of this germ theory, and also to put before you those diseases in which the germ has been found to be the actual cause of the disease. As far back as the year 1828 Ehrenberg found and described small organisms in water and dust, which he called "infusion animals." In 1837 Schwan stated that the air was laden with these germs, and that they were the cause of fermentation and putrefaction, and from that time the subject of micro-organisms has been taken up by many observers in all countries. Schwan was, in fact, the founder of the germ theory, and his original experiments were made with yeast, which he found caused, by its multiplication and vegetation in a fermentable fluid, this process of fermentation. This was followed up shortly by the discovery that if the yeast cells were broken up and their vitality destroyed, no fermentation took place, and also by the discovery that different forms of organisms produced different forms of fermentation. It has since been proved that germs are present in all fermenting and putrefying fluids, and that these latter can be kept from fermenting or putrefying if the germs are by some means or another excluded. This exclusion of germs may be done in various ways. As far back as 1836 it was shown that if putrescible substances were boiled, no decomposition took place if the access of air were then prevented.

The air may be heated, or filtered through cotton wool. Now these experiments are important, as on them are based all the principles now used for the preservation of meat foods, &c., for lengthened periods. Tinned meats and fruits are heated, the air almost totally expelled, and then the tins are hermetically sealed. Mutton is brought from Australia and New Zealand frozen, and quite fit for human food. These examples might be multiplied very largely, but enough has been said to show the reason why freezing in the one case, and heating and exclusion of air in the other is had recourse to.

It has been abundantly proved that these organisms which cause putrefaction are present everywhere, in air, dust, water, and earth, the air being the most common carrier.

Of more importance to us, however, are those micro-organisms, which are the casual agents in the various zymotic and contagious diseases. It has been definitely proved that in some diseases the organism is the direct cause of the disease, while in a large number of others, it is only by analogy that we can say this is possibly the case.

In no case can it be said that a disease is directly caused by an organism unless (1) the micro-organism is found in the blood or tissues of the man or animals, dead of the disease or suffering from it; (2) unless the organism taken from the animal can be cultivated on nutrient media for several generations; that is to say, a tube of, say, sterilised blood serum is inoculated with the germ, then put into an incubator at a certain temperature, and when the growth is active, this is again inoculated into another tube of sterilised blood serum, and so on for several generations; (3) the growth from these tubes ought, if introduced into a healthy animal, to produce the same disease as the one from which they were first taken; (4) the same organism should be found in this new animal so inoculated.

The organisms in which we are interested, viz., those which produce disease, belong to the lower fungi, a branch of the vegetable kingdom which are propagated by means of spores. These fungi are divided into four groups: (1) The true fungi, or mould fungi; (2) Mycetozoa; (3) Yeast fungi, or Blastomycetes, and (4) the fission fungi, or Schizomycetes.

It would be beyond my province, even if it were altogether necessary, to enter into a minute description of these divisions, but it is necessary to mention the different varieties of the mould fungi, which are interesting from a hygienic standpoint.

(1) The blight fungi (Ustitago). These are parasites on different kinds of grain, as wheat, barley, and oats; they form a black powder on the ears and panicles of these grains.

(2) Empusa muscae, a parasite of house flies, very common. Flies dying of this are seen to have three white belts between the segments of the posterior part of the body.

(3) Fungus of potato disease.

(4) Fungus of ergot.

(5) Rust fungi, or Puccinia graminis.

(6) Aspergillus. There are four or five different kinds of this. These are the moulds one so often sees on bread, jelly, old boots, &c., and lately it has been found that some of them can grow in the blood of mammals. All these fungi are very widespread. Some of them grow at one temperature and some at another, so that by raising or lowering the temperature the different forms can be cultivated.

(7) Oidium, or mildew, attacking plants, but oidium lactis may be found as a whitish coating on milk or bread. It was at one time thought that the different forms of ringworm were produced by this last fungus, but this has been found not to be the case, although that disease is caused by a fungus, closely allied to oidium lactis, but differing slightly from it as to the temperature at which it can be cultivated.

(8) Mucors, found on putrefying substances as white or brown patches.

(9) Penicillum, or pencil mould. The most common of all moulds.

II.—The Mycetozoa. There are none which are directly the cause of disease. They live chiefly on dead vegetable matter, although they are also found as parasites on the higher plants and occasionally in the animal body.

III.—The Yeast Fungi. These multiply by what is called budding. Each yeast cell multiplies by means of projecting bud from the original cell, which grows larger and is then detached from the parent cell. To this class belong all the kinds of yeast, the germ existing in ordinary vinegar, and the fungus of thrush, a very common disease in children, especially when hand fed. There are different kinds of yeast: (1) Yeast producing beer; (2) yeast producing the spontaneous fermentation of wine; (3) that found in acetic acid; (4) pink-coloured yeast. This is very common.

IV.—The fission fungi, Schizomycetes; also called microbes and bacteria. These are what are ordinarily called germs. I do not wish to enter into any detail regarding the many varieties of this form. It will be sufficient for our purpose to mention the main divisions, and then to describe the principal forms in each division, especially those forms which are the cause of disease in man.

(1) Micrococcus. These are just like round or oval dots and they possess no power of movement.

(2) Bacillus. These are rods in which the length is from two to four or more times the breadth.

(3) Spirilla. These are twisted threads and are nearly all moveable.

(4) Bacteria of irregular forms.

### Nursing in Workhouse Sick Wards.

We have received from the Local Government Board a memorandum dated April, 1892, which deals with this important subject so clearly that the most obtuse guardian cannot fail to see its importance, either in its relation to the humane part which our guardians are meant to play, or to that other momentous question, the saving of the ratepayers' money. The memorandum gives the circular letter which the Poor Law Board issued in May, 1865, which gave as clear a description of the undesirability of pauper inmates acting as assistant nurses as anything which could be written now, when the abuses which follow such management are fully recognised. The necessity of going over old ground because law is allowed to become a dead letter is disheartening and wasteful, and the circular letter of 1865 is an excellent example of this; the Poor Law Board then spoke as strongly as possible of the need of trained nurses adequately remunerated, and recommended that guardians should, "as far as possible," discontinue the practice of appointing pauper inmates to act as assistant nurses in the infirmary or sick wards. Space forbids our giving in detail the recommendations in the new memorandum, but next week we hope to deal exhaustively with it.

### Rural District Nursing and the Jubilee Institute.

Sir,—May we hope, through your kindness, to draw attention to the work carried on by this Association? It has for its object the trained nursing of the sick poor in their own homes in country districts. During the late visitation of influenza, in many country villages whole families have been prostrated by the disease; and it can easily be imagined in such cases how terrible was the condition of the sick, who were sometimes wholly unattended and without the comfort of proper food or nursing. In the comparatively few country places where a trained nurse was established there was some one to go from house to house; carrying out the doctor's orders and doing what was possible for the relief of suffering and the promotion of recovery. But generally no such help was available; and though the epidemic has drawn special attention to the importance of trained nursing, we have ample proof that it is at all times of scarcely less importance among a rural population often remote from medical help. May we hope, therefore, that those who have experienced the comfort of good nursing in serious illness, in confinements, in cases of accident, and in epidemics such as the recent one, will help us to extend this blessing to their poorer brethren of the labouring classes, to whom the loss of health often entails the loss of the means of subsistence as well?

The Rural District Branch is importuned for nurses; but the supply of those thoroughly trained and suitable for country work is not nearly adequate to the demand. The work of the branch in training nurses to supply this demand, and in supplying nurses to districts, in many cases at a smaller charge than the actual cost, has rapidly extended during the last twelve months. The Committee are now training, wholly or partially, twenty-two nurses, all of whom are under engagement to undertake rural district nursing as soon as their training is completed; and they appeal to the public for funds to enable them to continue and extend this work.

The necessity for funds for this very important purpose will be better understood when we state that to train one nurse fully takes about two years and costs over £50; and if we had the means of training ten times as many as we are now doing we should scarcely meet the demands made upon us. The fund of the Rural Branch is entirely distinct from that of the Queen's Institute, from whom, however, we receive a subsidy of £250 per annum.

Donations and subscriptions (especially the latter) will be thankfully received by either of the Treasurers—Sir John Lubbock, Bart., M.P., 15, Lombard Street, or G. E. Martin, Esq., Old Bank, Worcester; or by the Secretary, at the office, 12, Buckingham Street, Strand.—We are, sir, your obedient servants,

(Signed) K. Westminster. Victoria A. Lambton.

May 10th. Maud Wolmer. Lucy C. Hicks Beach.

HEAVENLY LOVE.

When our hearts are full of love for an earthly object, the first thing we desire is for that love to be returned as soon as we are sure of it, our happiness seems complete, perfect, and certain. Alas! man is a changeable being; sometimes it is we who alter our minds, at others, the object of our affections changes his or her opinion. Whither then has our happiness fled? Shall we not take it as a warning that no earthly affections can ever satisfy or be lasting? It will be wise so to do.

But we must have some warmth, some sympathy, in our lives, or we become mere machines, working on day by day in a grove of selfish, lifeless drudgery. Out of this gloomy world which we are making for ourselves we can only escape in one way, and that is by returning the love of Him who is yearning to gain it. The Father who loves us so dearly that He gave His only Son for our redemption, the Son whose equal love led Him to shed every drop of His precious blood for us, the Holy Spirit who melts with His breath the icy numbness of our hearts—if we will respond to Him; all are full of love for our bodies and souls, and long for this affection to be returned. The Father says, "Come, My children, return unto Me, your sorrows and sufferings are but sent to draw you back to My bosom from whence ye have wandered, the fatted calf shall be killed, the robe and the ring are only waiting for you to take them. If your sins are as scarlet they shall be white as snow, if they be crimson yet shall they be like wool. Return then, come." Our dear Lord says, "Have I not shown you how I love you by my woes, by my sufferings? If you will but love Me I will bear your griefs and carry your sorrows; you shall no longer be weary and heavy laden, for I will refresh you." The Holy Spirit whispers to our hearts in tones softer and sweeter than the early morning breeze, "Love Me, and I will fill you with all peace and joy in believing."

How can we be regardless of such invitations from a God who watches over our lives and leads us with blessings more than we desire or deserve, who gives His angels charge over us to keep us in all our ways. He does all this even when we only shun Him, but if we pour out our whole hearts before Him, He will increase His blessings fourfold. The parched tongue, the aching head, shall be cooled by the rivers of water which flow from the throne of heaven; the trembling limb, the aching heart, shall be strengthened by the fire of His love. For our love once set on a divine image can never fail; the more we think of Him the deeper will our happiness become.

"God only knows the love of God;
O, that it now were shed abroad
In this poor stony heart.
For love I sigh, for love I pine;
This only portion, Lord, be mine,
Be mine the better part."

### Among the Institutions.

The **Hanley Nursing Society.**—These nurses paid 5,264 visits during the year. We are sorry to see that funds are not in the flourishing condition they might be.

**Conway District Nurses.**—Nurse Kate Jones started the work of this association in July, 1891, and up to now she has paid over 2,000 visits. This nurse started under great difficulty in the shape of local prejudice against her profession, but steadily one case has led to another, and she has quite overcome all opposition.

**West Hartlepool District Nurses.**—The second year's work sees a second nurse at work; 2,969 visits were paid during the year, and finances are very satisfactory, the year beginning with a balance in hand of £88. Fourteen ladies have been enrolled as associates of the St. John Ambulance Nursing Guild, and they assist the professional nurses in whatever small details they are capable of.

**City of Cork General Hospital.**—We should be very glad indeed to hear of a few cheques and postal orders wending their way to our sister island. Money of any sort seems scarce there, and money in hospitals still scarcer. We hear that £731 is weighing on the minds of those who work at this useful institution; and, in fact, money is wanted for the actual carrying on of the work. The nuns work faithfully and zealously in their nursing of the sick in this hospital, and we much regret to hear of anxiety as regards finances; here is another opportunity of doing good for any rich man or woman who wants to enjoy the pleasure of giving.

**Camborne District Nurses.**—Perhaps nowhere in England were the services of a district nurse more required than they were in the parish of Camborne, in Cornwall, three years ago. This large industrial centre, with a working population of 14,000, has neither hospital nor infirmary; the majority of the men are miners, while the unmarried women and girls work on the tin streams and tin floors of the large mines. The nurses paid 4,682 visits, and have been most devoted and untiring in their work. A few more subscriptions would be more than welcome, as there was a deficit of £12 at the start of the year. Perhaps this notice may catch the eye of somebody who would be glad to wipe the debt off.

### Where to Go.

The Royal Academy.—We noticed one plain nursing uniform among the crowd of celebrities which gathered at the private view on Friday last, and the neat and graceful usefulness of this attire struck us more strongly than ever. One's chief impression of the present fashion was that trains are only made to be trodden on. Pictures of interest to the nursing profession specially are with one exception absent this year; Mr. Dering Curtois takes us to Johnson Ward, Lincoln County Hospital, and looking at it critically, we thought the details accurate, and the ward a decidedly cheerful one as far as sunlight goes! There is a picture called "The Amateur Dentist," by Ralph Hedley, which has a certain melancholy interest; a little boy is having a small tooth extracted with the familiar piece of string; it reminded one of schoolboy days and primitive dentistry generally. "Bonnie Prince Charlie" is a picture which we hope our readers will look out for. The Prince stands a little forward with flowers and ribbons strewn at his feet, a little to the back stand two chieftains whose anxious faces seem to give the keynote of coming doom; it is a beautiful painting which tells its own story. "June in the Austrian Tyrol," and "Over the Sunlit Sea," by Mr. MacWhirter, are two of the loveliest pieces of colour in the exhibition. A portrait of Mrs. Frank Grimwood wearing the Royal Red Cross will interest our readers, and "Forging the Anchor," by Stanhope A. Forbes is another picture, one of the few we can mention, which is one of the most striking, the heat and glow of the red hot iron, and the muscles of the men's arms who are raising the great hammers, are splendid studies. And here we must end our short notices of the pictures. Criticism is not our province, nurses must go and see, and approve or disapprove for themselves; there is no better relaxation than an afternoon spent among the canvas people and lands at the Academy.

The Southwark Picture Exhibition will be open in the rooms of the Polytechnic, Borough Road, from May 28th to June 19th.

Miss Angela Vanbrugh, the talented young violinist, promises music lovers a treat at her concert, to be held on June 10th, at three p.m., in the Prince's Hall, Piccadilly. The programme, which is an excellent one, will be carried out by Miss Evangeline Florence, the soprano who possesses a voice of such wonderful compass that she goes under the name of "The Eiffel Note," Madame Marion Mackenzie, Mr. Barrington Foote, and other vocalists, and the instrumentalists include, besides Miss Vanbrugh, Miss Dora Bright, and Mons. Hollmann. Tickets can be had at the hall, ranging from 1s. for admission, to 10s. 6d., the price of front stalls.

### Everybody's Opinion.

*[Correspondence on all subjects is invited, but we cannot in any way be responsible for the opinions expressed by our correspondents. No communications can be entertained if the name and address of the correspondent is not given, or unless one side of the paper only be written on.]*

### CORK AND ITS NEIGHBOURHOOD.

"M.|T. S." writes: Having read "A Lover of Nurses'" suggestion about spending a holiday in the south of Ireland I am rather piqued at the writer saying "Cork is not an attractive place." If the city is not attractive, there are many places, very easy of access from it, rich enough in beauty of scenery to revive the tired mind as well as the body. Who has not heard of "The Groves of Blarney." Should a tired nurse (and I know what that means), wish to stay for a few days in our southern capital, it will give me great pleasure to give her every assistance. Rooms may be had for six or seven shillings weekly, bread, butter, and eggs are cheap and very good. The cross channel boats are most comfortable. I should be obliged by your giving my address to any nurse requiring information about Cork or its neighbourhood.

### THE LOWESTOFT HOSPITAL.

Mr. Frederick Morse, the Treasurer, writes:—In your paper of the 7th inst. you say: "We are sorry to hear of troubled waters at this institution." You will be glad to hear "there is smooth water for us on the horizon." I should not trouble you by answering anonymous letters if your notice had not had the effect of hindering applicants for the Matron's situation from accepting our invitations; your informant evidently wishing to injure the institution, has made an insinuation "that the Committee have tampered with the rules." I deny this *in toto*; and I may further add that the insinuation is made without the sanction of the gentlemen named. In the early part of 1877 we had only one Sister to attend to both the male and female wards. The surgeon requested us to have two Sisters, one for each ward, and which request the Committee acceded to at that time. At the present time the Committee have backed up the report of the surgeons, not tampered with the rules. I have watched the progress of the Lowestoft Hospital for nearly half a century, and I can testify to the excellent and useful work that has been carried on at that institution. With the assistance of our three most excellent surgeons, and smooth water on the horizon, a prosperous future is in store for the Lowestoft Hospital.

### Experiences of a Trained Nurse in Valparaiso during the Revolution.

*(Concluded from page xlix.)*

THE day's routine was somewhat like this: tea and roll at half-past six a.m.; at half-past eight the doctor would make a hurried round; by ten we tried to have the ward and patients as tidy as possible; eleven was the patients' breakfast hour; this was a substantial meal consisting of soup, a mess of beans, potatoes, and meat, or meat and a kind of seaweed of which the patients are very fond, also rolls and tea, or the thin claret so much drunk here. At two o'clock they had a cup of milk or tea and a roll, and at half-past two the doctors came, saw the wounds dressed, and did operations, and we were always glad if they left by five, the patients' dinner hour. This meal generally consisted of soup, roast or stewed beef, with maccaroni or rice, and stewed fruit. At eight the doctor usually paid his last visit for the day. The day and night attendants came on duty at seven a.m. and seven p.m. respectively.

The worst cases we had were brought from Concon, in the steamer "Aconcagua" nine or ten days after the battle, and their wounds were in a very bad state. The history of one case might stand for all. Poor S., whose father was an Englishman, had enlisted when the war broke out; he was shot in the left leg below the knee, the bone being shattered. He said "We were going up a hill at Concon when I suddenly fell; I felt nothing till my leg began to bleed, and then it did hurt me. Next day some men came and robbed me of everything except my shirt and trousers, they even tried to pull the boot off my broken leg, and then, oh! I did scream, and begged them rather to kill me. I lay out on the hill for four days, and it rained, and I was so cold and had nothing to eat except one roll; then some soldiers found me and took me to San Pedro, where they put my leg in a splint. I was then brought by sea to Valparaiso in the "Aconcagua," it was terribly overcrowded and we suffered very much. On arrival I was taken, with many others, to San Augustine (the Government Hospital), but as every bed was occupied we were laid in a corridor all night and brought here next day, and oh, no one knows what we suffered." On this man's admission, both bones were found to be fractured, the leg was swollen and of a dusky red, with discharge pouring from the wound. Amputation below the knee was performed on September 3rd, the wound being dressed with iodoform gauze. Notwithstanding all this patient had gone through he made an excellent recovery, the wound being healed by September 20th, and his temperature never at any time rising above 101 deg.

Many of the men seemed like the proverbial cat with nine lives, so much had they gone through; one of the patients who was wounded at Placilla was brought to the hospital with a fractured femur; the ball (a Mannlicher) had entered the left buttock and taken a downward course, fracturing the femur; he had also a large wound in the right thigh, caused by a fragment of shell. He was at first sent to the ward which was under the charge of American surgeons from the "San Francisco" and "Baltimore," and had amputation of the left leg at the hip, but the wound did badly. The American surgeons being soon obliged to leave, their worst cases were drafted into the other wards, and A. came to us; he was, poor fellow in a very bad state, wounds in both legs, sloughing and very offensive, with a large bedsore; he was continually crying out for opium to relieve his sufferings. I put him under the special charge of a lady and told her to feed him up in every possible way, the result of her care being very soon apparent. On September 19th, disarticulation of the head of the femur was performed, and two large drainage tubes introduced, one communicating with a wound in the back. October 11th, patient better, bedsore healed but discharge still offensive. October 15th, he had severe hæmorrhage from sloughing of femoral artery, and the wound which was healing had to be opened and the artery ligatured in two places; patient was much collapsed, pulse could not be felt. October 20th, wound in right leg discharged, and on introducing a probe, what seemed to be a piece of bone could be felt; the opening was enlarged and two fragments of shell, one an inch and the other about half an inch in length were extracted. October 22nd, wound in right leg healing; patient wasting, temp. 100 deg. to 104 deg. This patient gradually improved, and was sent to the San Augustine Hospital on December 1st, when the ambulance was closed. When I last heard of him he was able to walk with crutches, and looked fat and well, and reported his wounds as being almost healed.

We had some very successful cases of lung shots, the only poor fellow who did badly was an officer who had been shot through the right lung just below the clavicle. He came to us with inflammation of both lungs, due no doubt to exposure; he was taken home on September 26th, the wounds had healed but there were signs of commencing phthisis. It was interesting to notice the way in which pieces of clothing were carried right through the lung by the bullet forcing them before it; unfortunately, small pieces were often left behind, setting up inflammation and keeping the wound open till they came near enough the surface to be pulled out with forceps. The wounds from shell are the most painful to see; the shell injures such an extent of surface, tearing away skin and flesh in a horrible manner; one man had the front of his leg from knee to ancle torn away by a fragment of shell which had gone so deep in one part as to nearly divide the tibia; another man had all his fingers torn off one hand leaving a ghastly bleeding stump. The Mannlicher is certainly the most humane kind of bullet as it goes clean through, inflicting a much smaller wound than the old-fashioned bullets with which the Government troops were supplied.

In the ambulance we had 244 cases, of these 29 died; 138 operations were performed, 40 being amputations, with 14 deaths; the dressings used were the ordinary iodoform gauze or sublimate gauze, latterly Pyoktannin was used for dusting the wounds, with good results. We had to exercise great economy in the use of dressings, as the supply threatened to run short, a small mackintosh for use during the dressings was a valuable possession, there being none to be bought in Valparaiso after the first fortnight. My ward was emptied on November 1st, as the house it was in had to be given up to the hotel proprietor, and the rest of the Ambulance was closed on December 1st, those patients who were unable to go home being sent to the San Augustine Hospital. I could not help admiring the way in which the ladies of Valparaiso came forward to help in the work, I could not wish for better amateur helpers than the ladies who worked in my ward; no work seemed to be too hard or disagreeable for them to do. Dr. von Schrœder presented all those who assisted at the Ambulance a little silver medal, having a red cross on one side, and the date of the Battle of Placilla on the other.

### Notes and Queries.

### Queries.

*An Anxious Learner.*—Where can massage be learnt in Birmingham?

*N.P.*—Can anyone give me patterns of Hospital Flannel Jackets for men and children as soon as possible?

### Answers.

*Edith.*—Certainly; go to the infirmary you name if you have the chance, or you could apply to any hospital you see advertising for probationers, and mention your height. I would advise you to think well before leaving such a good situation as you have; there are difficulties to contend with everywhere.

*F. C.*—Your first question can only be answered by the Lady Superintendent. 2. Uniform is provided. 3. You could read Lewis' "Theory and Practice of Nursing." Train thoroughly when you once begin, and write again if you want more advice; it is no trouble, thank you.

*Heather.*—1. Guy's, St. Mary's, or Middlesex give them. 2. Certainly return to your old hospital if you can, and do not be content till you have gained as good a certificate as they give; they are as good as the others you name. 3. You may mean the Nurses' Residential Club, 92, Charlotte Street, Fitzroy Square.

### Matrons.

The term Matron is usually associated in our minds with the idea of a person holding office in a hospital, workhouse, school, or other institution. Sometimes she may be a harsh-voiced, hard-natured, forbidding-looking woman, who maintains discipline by fear alone, and though, doubtless, obeyed in all outward ways, her severity begets cunning and deceit in those who serve under her. One shudders to think of the almost unbounded power which has, at various periods, been left in such ignorant hands. To her stands in pleasant contrast the bright, good-tempered woman, who, whether wife, widow, or spinster, possesses above all things that great gift of motherliness which endears her to all young and to all suffering people. Her native sympathy with, and affection for her fellow creatures, give her an amount of influence which no mere culture or refinement alone could ensure.

The nature of the duties now required from a matron of an institution vary according to the size and character of the establishment, and we still find in some of them the plain and homely housekeeper, whilst in others an elegant lady in a rustling silk dress, represents this official, and somehow strikes us as being rather of the ornamental, than of the useful type.

The matron in a high-class school is often a lady of refinement and sound judgment, and many are the boys whose first impulses towards manly self-reliance as well as of deference to all women date from the school days when "Matron's Room," formed in their eyes, a sort of oasis where, although sure of sympathy at all times, no mean or cowardly acts would be condoned, whilst all faults, if confessed, would be as freely pardoned as if their own mothers were their judges.

In workhouse infirmaries we are thankful to see, slowly but surely, the trained nurse superseding the untrained matron, and our "unfortunate" brothers and sisters at last are coming to be treated with merciful skill. These trained matrons are still distressingly hampered by the very limited number of nurses allowed them, and we long for the day when our poor shall be more sufficiently tended. "Infirmary cases"! Yes, this is a term full of significance; it means, speaking generally, those heavy chronic cases of illness passed on from general hospitals as unsuited to their wards, where they have already perhaps remained for a period exceeding the nominal limit of time. It also means patients, far gone in consumption, whose friends possibly objected to parting from them at an earlier stage of the disease, but who are now, through pressure of poverty, obliged to pass on the poor creature who has engrossed so much time, and who needs nourishment which they can no longer supply. When we think of the care that such a person would need and obtain in a prosperous home, we shudder to know that a pauper gets but one-sixtieth share of a night nurse's attention, and this supplemented only by such grudging help as his poor fellow-sufferers will bestow. Almost worse is the case of the paralysed patients, who are literally more helpless than babies. However, a good beginning, in the right direction, has been made in each workhouse infirmary where a fully-trained matron has been placed at the head of the nursing department, and a better supply of efficient workers will doubtless be granted by-and-bye.

The hospital matron is a personage of very special interest to most of our readers. Happily the doubtful title, "Lady Superintendent," is nearly obsolete, for we fancy it appertained to the days when untrained ladies held these posts, and perhaps it was as descriptive a designation of that order of things as could be found. Of course, a few of these untrained ladies still hold these important positions, but they are getting more rare year by year, and we can therefore anticipate the day when such anomalies shall no longer exist.

If nurses are to be taught and trained efficiently, it is necessary to place over them a lady who knows more than themselves, not only theoretically, but practically. Unless she has gone through a thorough training herself, in day and night nurses' duties, she cannot be considered a competent matron, for she is unable to judge accurately of the relative value of details, or to estimate fairly the amount of work which each nurse or probationer should have expected of her.

With the best intentions in the world, she cannot be just in deciding points of which she has practically no knowledge. She may pick up opinions from other people, but "opinions" must not be confounded with experience, and a matron who is driven to form her judgments on hearsay is no fit ruler of women.

Alas! Women do not always govern well, they are too often moved by impulse, by personal feelings, by instinctive bias towards the first side of a question presented to them, by a number of small considerations which do not affect the calmer, if slower, male mind. Sometimes a too sensitive conscience is a hindrance to impartial government, and again a less-sensitive one is a snare.

Above all other qualities, a good matron aims at strict justice. She never decides a question against an absent subordinate; but on all occasions gives opportunity for explanations to be offered by the accused, whether the fault be small or great. The ideal matron is, firstly, a good woman; secondly, a wise lady; and thirdly, a perfect nurse, and one who will never rest contented until her training school is as celebrated for its high moral tone as for the perfection of the teaching.

Of the type of woman who combines flirtations with the serious work she professes, we have as little to say as of the perfectly "proper," but narrow-minded one, whose pettiness excludes all sympathy with those whose natures are greater than her own. Both are harmful as guides, and the latter makes more enemies than the former, for the flirt is generally pleasant, and therefore has a certain popularity even amongst her critics. The agreeable "woman of the world," although no true nurse, is always a favourite with strangers, but those who work under her would give a less satisfactory verdict on her influence.

The matron whose work holds the first place in her thoughts, who lives amongst her nurses, taking a kindly interest in their "play" as well as in their work and in their personal comforts; feeling sympathy with their weakness and failures as well as pride in their successes, will be served faithfully and lovingly, none the less because all know that she never lowers for an instant that high standard of excellence which she is bent on attaining for them and for herself.

### Technical Teachers.

A writer has lately pointed out in the *Queen* that since the County Council has been made responsible for the teaching of domestic science another opening for women workers occurs. There has been some difficulty in obtaining qualified teachers, and some of these after having been only a very short time preparing for an examination in their subject are at once enabled to gain a salary of £100 a year and upwards. It may sound a good deal more important to be a high school mistress, but considering the number of applicants for a post of any sort nowadays, it would be well for young women to turn their attention County Council wise. This lecturing would be a delightful occupation to a woman who thoroughly knew her subject, infinitely to be preferred to the tedious existence of many a private governess, and the scope of the work will quickly grow larger.

### Four Months in a Hospital Ward.

### A PERSONAL EXPERIENCE IN A PROVINCAL HOSPITAL—III.

### The House Doctor's Visit.

The House doctor is a punctual man, and at nine o'clock to the minute we hear his step in the corridor, and with a most refreshing absence of anything in the shape of affected wisdom or importance, he makes his way to the first bed, followed by the charge nurse and two probationers—quite a little procession. The doctor stands on one side of the bed, the charge nurse on the other, and the two probationers at the foot. "Better this morning?" he asks with a pleasant smile, and then patiently listens to anything the patient may have to tell him: only a sentence perhaps, some fancied fresh symptom of no import, yet the doctor is never hasty. I wonder if he knows how many hours of thought that one sentence has cost the patient, and how eagerly the man has looked for this moment to unburden his fears, or to get confirmation of some ray of hope he thinks he sees. I am sure the doctor must know all this, for he never ridicules the poor fellows, and we all know what foolish fancies will crowd into a sick man's brain. Then the doctor would question the nurse as to nursing details, and sometimes he would make an addition to his cabalistic signs on the "bedboard," or order a hot bath, or a painting, or blistering; sometimes in a low voice, sufficiently loud for the patient to hear, he would say "Very satisfactory," or some such short encouragement, and sometimes he would pass on without a word, but I used to think I could see looks of intelligence between him and the charge nurse which silently spoke of waning hope. To some happy mortals he would give practical proof of their convalescence by ordering them to "sit up," and to others still stronger proof in a promise of "discharge" next "visitors' day," and the glad faces of those lucky ones showed how precious to them would be this certificate of restored health. Visions of wife and children waiting all these weary weeks while the "bread-winner" came not, sad and lonely at the best, but still more sad when the terrible doubt hung over that hearth, Will he ever come back? Visions of wife and children, vivid and real, passed before their eyes, even though it was broad day, and from their heart of hearts went out a silent thanksgiving for the mercy of this great deliverance. The doctor has now seen us all, and passes into the clinical room with the charge nurse. In a few minutes he is out again, makes further additions to one or two of the "bed boards," and we hear his retreating footsteps as he goes along to some other group of the lame and halt gathered under the roof of this God-sent institution.

### A Babel of Voices.

The ward, hushed and still as the grave while the doctor was present, now breaks out into a Babel of voices. The sympathy between these seventeen invalids, strangers all of them until they met here, is something wonderful, and later on I will try to give you a few instances that came under my own notice of that "brotherly" love which, whatever they may have been outside, they displayed to such a remarkable degree during their sojourn in this medical ward. At half-past nine o'clock luncheon was served out—half-a-pint of bread and milk to each man—and it was left to our choice as to whether we would have it cold or hot. Some preferred the bread boiled with the milk, some the bread put in afterwards. All these little preferences were studied by the nurses. Now the charge nurse and her probationers were moving swiftly about in execution of the doctor's nursing directions, and in the taking of temperatures, this last detail being repeated frequently in certain cases throughout the day.

### The Matron's Round.

Then came the Matron on her morning round of inspection—a tall, graceful lady of exquisite carriage, a trace of suffering in her face, I thought, but yet with the sweetest expression I ever saw. Her manner towards the patients, for each of whom she had a pleasant word, was rather that of an exceedingly amiable patron than of a salaried official. It did one good to look at her. Again, the demeanour of the nurses shows us that some one of importance is coming, and there enters the House Doctor, followed by a tall, thin, elderly gentleman, with a grisly beard, and slightly stooping, of whom it needs not the bright metal stethoscope, sticking half-way out of his pocket, to tell us that he, too, is a doctor, for in his outline, his gait, and in his quick searching eyes he is the very personification of a wise old physician. Yes, this was one of the two Honorary Physicians attached to the hospital, who divided the patients between them, and attended on alternate days.

### A Large hearted Æsculapius.

For thirty years he had held his appointment, and during the four months it was my lot to be an inmate of this institution, I saw and heard enough to enable me now to say that a better man never lived than this large-hearted disciple of Æsculapius. He was in the "sere and yellow,' yet in all matters appertaining to the hospital, as zealous as a youngster but just in receipt of his diploma, and for all his great local eminence in the profession, he was as courteous as if in his own consulting-room, where guineas were cheerfully paid for no more service than he rendered gratuitously to the poorest of us here. I saw him come even on a Sunday afternoon to our ward to try to save a poor fellow who was dying, and I have known this generous man give money out of his own pocket to help a convalescent get comforts he had prescribed, after leaving the hospital; and again to help some fevered wreck to pay his fare to and from a convalescent home at the sea-side. Never tired of a case, never at his wit's end, always fighting the disease, to the last moment he never despaired, and to know that one's treatment was under his guidance was to know that one might fall fighting, but that the citadel of one's life would never be surrendered. My own "bed board" bore this gentleman's name, and in fear and trembling I waited his coming, and when he was gone I asked the charge nurse what he thought of my case, but she was far too well skilled in the art of nursing to betray the doctor, and so she gave me a clever answer that said nothing. The consultation between the Honorary Physician and the House Doctor was so extremely technical that I could make nothing of it, and so after the first attempt to gather information in that way, I let it alone, and upon my word I soon felt the easier in mind for doing so.

### Final Flight of all Wrong Impressions.

It was now half-past eleven o'clock on the second morning after my coming in, when the arrival of two new patients, looking pale and frightened, reminded me of my own entrance, and that I had now completed my first twenty-four hours of hospital life. How different it all was to what I had expected, and how ashamed I felt that I had ever given credence to the popular errors about the ravings and the groans and the curses and the scenes that have to be encountered in a hospital ward; much there is of grievous sorrow, much of the darker shadows of life; it is an experience which must leave its mark deeply engraved upon the mind of any man, however callous, who has gone through it, but not grievous in the vulgar sense of the term, for here you will see a calm patience and fortitude under well-nigh overwhelming pain and misfortune that are positively heroic.

(*To be continued.*)

### Tired of Life.

"Oh, that I had wings like a dove! for then would I flee away and be at rest."

"Let me die, doctor; oh, let me die!" These were the first words, uttered in a faint and indescribably pathetic voice, which fell upon my ear as I softly entered the bed-room of a new "case" to which I had been hastily summoned that afternoon.

I knew very little of the patient, except that she was a young lady who, at the early age of eighteen, had left father, mother, and home in England in order to share with her husband the uncertainties and vicissitudes of life in South Africa. This was all I had heard, except the intelligence, received in a hasty note from Dr. Wilmot, that she had been suffering for a fortnight past from the gastric form of influenza, and was in need of more experienced nursing than her husband or neighbours could give.

"Let me die, doctor; oh, let me die!"

How strangely hopeless was the tone, and how pitiful the expression of the white, worn-face of the speaker, as she lay inert and helpless on her bed.

"Let you die, indeed," said Dr. Wilmot in his cheeriest voice. "I should think not. Why you have years of life and happiness before you yet," patting as he spoke her nerveless hand.

The patient moved her head slowly in dissent, but said no more; and as Dr. Wilmot here caught sight of me, a smile of satisfaction crossed his face, while he said—

"Ah, here is someone who will know how to take care of you. We shall hear no more about dying now. A little good nursing, and we shall have you up quite strong and bright again."

After a few general directions, the doctor left the room, and I quietly followed him in obedience to a slight gesture.

"She is very low," he said, when we were out of hearing. "All the acute symptoms have succumbed to treatment, but have left her in a very weak, depressed state. That is why I wanted you here. You must do your best to rouse and stimulate without exciting her."

It was thus on very slender data that I commenced the nursing of my patient, and yet an hour or two was sufficient to make me feel that I had never had a case about which I longed to know more. Mrs. Daley appeared to have everything which youthful wives most desire. Her house, though not large, was very prettily situated on the skirts of the park, and was furnished with great taste and comfort. It was, in fact, one of the pretty homes now frequently met with in the colony, suggestive at once of a fond husband's solicitude, and of the thoughtful love of absent friends. She had one child, a beautiful, blooming boy, about a year old; yet all these things appeared to have lost their charm. Mr. Daley often came in to see his wife, and his evident anxiety proved his sincere affection for her. Yet even his presence failed to rouse her. It brought no animation to her face, or light to her eye. I even fancied that she seemed to shrink from him, and was easier when he had left the room. Yet I understood from one of their most intimate friends that they had always been devoted to each other. My patient suffered very little pain, and took uncomplainingly everything I gave her, but she rarely spoke unless addressed, and seemed utterly without a wish to recover.

I saw there was some mystery here, but kept my thoughts to myself, hoping that time would allow me to clear it away, or that returning strength would enable her to rise above it.

On the second day of my attendance, finding she still lay in the same passive state, I told Isola, the Kafir nurse-girl, to carefully dress little Bertie, and bring him to his mother's room, thinking this would surely rouse her, if anything could. When the child appeared in the arms of his dusky nurse, who was very good to him, he made a pretty picture in his white embroidered frock and blue sash. The little fellow clapped his hands gleefully, and shouted, "Ma—ma!" the only word he had learned to articulate. Few mothers could resist such an appeal. But Mrs. Daley glanced languidly at him, said "poor baby!" in a hopeless tone, and then turned away her head as if the sight of him were unwelcome. I signed to Isola to go away, but as she left the room I heard her say softly—

"Wow! Missis not want baby any more? Missis *very* sick then!"

This experiment having failed, I could no longer repress my anxiety, and on the next visit of Dr. Wilmot said—

"I am sorely puzzled over this case, doctor. My patient's malady seems to be more of the mind than the body."

"What makes you think that?" he asked, giving me a quick look.

"Because I cannot rouse her to take an interest in anything, not even in her child; and yet there seems to be no symptoms of illness sufficient to account for such depression."

"I cannot account for it either," said he. "I know very little more of Mrs. Daley than I have told you. I have only attended her since the birth of her baby. She may have had anxieties. I believe her husband suffered heavily when the depression in the gold market set in, and there may have been many difficulties over which she has fretted. The action of the heart is very weak; no organic disease, but very weak. However, her pulse is stronger to-day than it has been for a week past, and I hope we shall soon have her convalescent. You must try and get her spirits up, nurse."

This was indeed more easily said than done, but still I did not despair, and on the third day was encouraged by finding that she awoke brighter and more cheerful than she had yet been. It was Sunday, and in the afternoon she asked me if I would read to her the Psalms for the day.

Pleased with this request—for though I never like to force religion upon my patients, I am always glad when their own thoughts turn to it as a rest and refreshment in sickness—I found the portion for the 10th evening, containing one of the Psalmist's most pathetic appeals against the treachery of wicked men. When I paused she said—

"Ah, nurse, that does me good. Oh, that I had 'wings like a dove, that I might flee away and be at rest!'" repeating these words in a yearning voice.

"But your time for rest has not come yet," I said cheerfully. "You have probably many years of work before you yet for husband and child."

"I hope not," she said quietly. "I am tired of life."

"You only think that now because you are weak and low," I replied. "By and bye everything will seem different to you."

"No; I do not wish to live. Life has been too hard for me," she rejoined; then, as if fearing she had said too much, she added, "I think I could sleep now," and closed her eyes.

I did not like then to ask the question which rose to my lips, though more than ever I felt assured that there was some secret sorrow here which I longed to penetrate, and, if possible, remove, for a nurse feels herself heavily weighted when she sees that her patient's mind is oppressed by some hidden grief.

*(To be continued.)*

## Figures and Tables

**Figure f1:**